# The Invisible Suffering: Sexual Coercion, Interpersonal Violence, and Mental Health - A Cross-Sectional Study among University Students in South-Western Uganda

**DOI:** 10.1371/journal.pone.0051424

**Published:** 2012-12-11

**Authors:** Anette Agardh, Gilbert Tumwine, Benedict O. Asamoah, Elizabeth Cantor-Graae

**Affiliations:** 1 Social Medicine and Global Health, Department of Clinical Sciences Malmo, Lund University, Malmo, Sweden; 2 Centre for Adolescent Health, Royal Children’s Hospital, Murdoch Children’s Research Institute, Department of Paediatrics, University of Melbourne, Victoria, Australia; 3 Department of Obstetrics and Gynaecology, St Francis Hospital, Nsambya, Uganda; Federal University of Rio de Janeiro, Brazil

## Abstract

**Background:**

Despite a history of conflicts and widespread human rights violation in sub-Saharan Africa, little is known about the prevalence of interpersonal violence among the population in this region. Evidence from high-income countries suggests that exposure to violence has mental health consequences and violence also has associations with experiences of sexual coercion.

**Aims:**

This study sought to investigate the prevalence of physical and perceived threats of violence among university students in Uganda and to assess the possible relationship between such violence, sexual coercion, and symptoms of anxiety, depression, and psychoticism, respectively.

**Method:**

In 2005, 980 Ugandan university students responded to a self-administered questionnaire (response rate 80%) that assessed socio-demographic factors, social capital, importance of religion, mental health, experience of violence and sexual coercion, and sexual behaviour factors. Logistic regression analysis was applied as the main analytical tool.

**Results:**

Of those who responded, 28% reported perceived threats/threats of violence and 10% exposure to actual physical violence over the previous 12 months, with no significant gender differences in exposure history. Exposure to violence was significantly associated with the experience of sexual coercion among both males and females. Sexual coercion and threats/threats of violence were both significantly associated with poor mental health in males and females, but only males showed a strong association between exposure to physical violence and poor mental health.

**Conclusion:**

The current study suggests that in terms of general exposure, both males and females in the study population are equally exposed to sexual coercion and interpersonal violence, and both male and female students show generally similar mental health effects of exposure to such violence. The prevalence of interpersonal violence found in our study population may have long-term negative health implications. Our findings may serve as a baseline for interventions and continuing research aimed at preventing interpersonal violence.

## Introduction

Sexual coercion is a public health challenge with numerous consequences [1], [2] and predisposing factors [3]. Experiences of sexual coercion tend to be under-reported, although the consequences for its victims remain [4]. Previous studies have attributed this lack of reporting by victims of sexual coercion to the socio-cultural context of what people deem as acceptable or unacceptable in society [5] and also partly due to the unbalanced power relations between victims and perpetrators [1], [6]. Although generally overlooked in earlier years, sexual coercion is now increasingly gaining attention from the general public and the research community.

Sexual coercion is increasingly becoming a social problem among young people and university students [7]–[9] and has also been linked with exposure to physical violence, including threats of violence in this population [10]–[15]. Interpersonal violence including intimate partner violence has a huge impact on its victims [16]–[20]. Gender differences in exposure to physical or threats of violence remain unclear. Studies of exposure to interpersonal violence that include both males and females are rare in low-income countries. Some studies in high-income countries have suggested that women are generally more exposed to violence than men [21]–[23], while others contend that no gender differences exist [24]–[26]. Some studies that have linked physical violence to sexual coercion in young people concluded that victims of physical violence are likely to be sexually coerced [3], [27]. Others, however, also found that victims of physical violence are likely to practice sexually coercive behaviours themselves [28], [29]. Two large surveys done in the US and one among high school students in Norway and Sweden found that those who had been sexually coerced were two to four times at greater risk of forcing someone else into having sex [28], [30], [31]. In one of the surveys students who had been physically abused were more likely to be sexually coercive themselves [30]. Furthermore, Griffin and colleagues found that experiencing physical coercion or “incapacitated sexual victimization” was a risk factor for re-victimization, i.e. re-exposure to such events, with physical coercion being a stronger predictor [32]. These results corroborate findings from previous studies [13], [33]–[35], which concluded that in more than half of all cases young women have already experienced sexual coercion before they enter college [36], [37]. Therefore, many of these women are at risk of further exposure to sexual violence during their college years. As that period generally represents a transitional stage away from home, being in college contributes to the risk of exposure to several poor outcomes [32]. Moore and colleagues [38] theorised that the underlying mechanism for re-exposure to violence is emotional dysfunction resulting from earlier experience of sexual violence. They concluded that women who have previously been sexually coerced adopt strategies such as self-harm or risky sexual behaviour in an attempt to cope with the abusive experience they have had, thus exposing themselves to further risk of sexual coercion [32]. We previously found that 31.1% of students attending a university in Uganda had been subjected to some form of sexual coercion in their lifetime, with no significant gender difference [8], thus providing the current opportunity to examine sexual coercion in relation to exposure to interpersonal violence.

Moreover, studies across different populations have consistently linked sexual coercion to poor mental health [39], [40]. The findings from a WHO report also show that exposure to interpersonal violence is an important contributor to poor mental health [41], [42]. Given that exposure to violence is often underreported to the police or healthcare professionals, and that in most low-income countries, mental health care services are hardly accessible to the majority of the population, estimating the proportion of mental illness attributable to violence through the health care system is extremely challenging [16], [43], [44]. As a result, in the absence of epidemiological studies, a knowledge gap exists regarding the mental health consequences of exposure to violence in low-income countries.

In Uganda, various studies have been conducted concerning sexual coercion [5], [8], [45], mental health [46] and interpersonal violence [27] among university students and the general population. In the Rakai District of Uganda, adolescent women perceived sexual coercion as a normal part of intimate relationships, although participants in the study admitted that coercive sex reduces a woman’s quality of life and self-confidence by exposing her to risky sexual behaviours and re-victimization [47]. None of the studies mentioned above have investigated prevalence of interpersonal violence among university students and also none have investigated the possible association between exposure to physical violence (including threat of violence) and sexual coercion or their associations with mental health. A better understanding of the relationship between exposure to violence and mental health may facilitate the development of appropriate interventions and prevention strategies. The primary aim of this study was therefore to estimate the prevalence of exposure to threats, threats of violence and actual violence among young people in Uganda and the potential association between such experiences and experiences of sexual coercion. Our secondary aim was to examine the potential association between sexual coercion, threats, and actual violence and mental health status. Gender differences were examined throughout. Since social and economic conditions of poverty in low- and middle-income countries have been linked with common mental health disorders [48], our study also examined socio-demographic and lifestyle factors that could potentially modify the relationship between perceived exposure to violence and mental health.

## Methods

### Study Population

The study was performed at Mbarara University of Science and Technology (MUST), located in the centre of Mbarara, Uganda’s second largest city, approximately 350 km southwest of Uganda’s capital city, Kampala. Our target population consisted of undergraduate students from the university’s three faculties of medicine, science, and development studies. The sample comprised the entire undergraduate class of the university in 2005 (n = 1220 students).

### Data Collection

Data was collected by means of an 11-page self-administered questionnaire consisting of 132 questions that was distributed in lecture halls to all undergraduate students at MUST. Students were orally informed beforehand about the purpose of the questionnaire and were given instructions for filling it out. A consent form on the front page also contained a written explanation and justification of the project to be signed by the students as acknowledgement of being informed and agreeing to participate. Contact details for the principal investigator and a research assistant were provided, in case any questions or personal concerns would arise while answering the questions. While students were engaged in filling out the questionnaires, the research staff ensured that the room was silent so that each person could work in private. The consent forms and the questionnaires (without identifying information) were collected separately and placed in different boxes in the front of the rooms. A total of 980 students completed the questionnaires, representing 80% of the undergraduate students at the university. The research project was approved by the Institutional Ethical Review Committee at Mbarara University of Science and Technology.

### Study Protocol

The study protocol consisted of a self-administered questionnaire comprised of 132 questions assessing a range of items about demographics, lifestyle factors, social capital, sexual relations, behaviour, and self-rated health. The present study uses a subset of items regarding sexual coercion, exposure to violence, and mental health. The language of the questionnaire was English. All educational programmes at MUST are conducted in English.

### Socio-demographic Characteristics

Socio-demographic characteristics assessed were gender, age, place of childhood residence, and highest educational level attained by the head of the respondent’s family. For the purpose of analysis, age was divided into “younger” (≤23 years) and “older” (>23 years) groups, due to the greater risk of onset of most serious mental disorders prior to age 24. Student’s area of origin was classified as “rural”, “urban”, or “semi-urban/small town”. This variable was then dichotomized as “rural” and “semi-urban/urban”.

Family educational background was defined as the educational level the head of household had attained while the student was growing up, as follows: “did not finish primary school”, “completed primary school”, “completed secondary school”, “post-secondary school”, “college or university education”, and “other”. The variable was then categorized so that “did not finish primary school” and “completed primary school” were both coded as “low” levels of education, “completed secondary school” and “ post secondary school” were coded as “medium” levels of education, and “college”, “university education”, and “other” classified as “high” levels of education.

### Lifestyle Factors

Two lifestyle factors, cannabis use and alcohol consumption, were included in the analyses due to their potential association with experience of violence and with mental health and sexual coercion. Students were asked, “Have you ever smoked cannabis/marihuana?” and could answer: “No”, “Yes, during the last month”, “Yes, during the last year”, or “Yes, more than one year ago”. For analysis this was recoded into three categories: “No”, “Yes, during the last month”, and “Yes, during the last year or more than a year”. In response to the question, “How often have you consumed alcohol during the past twelve months?” students could respond: “4 times a week or more”, “2 to 3 times a week”, “3 to 4 times a month”, “Once a month or less”, or “Never”. This was then recoded into three categories: “Never” if the response was “No”, “Frequently” if the response was either “4 times a week or more” or “2 to 3 times a week”, and “Seldom” if the response was either “3 to 4 times a month” or “Once a month or less”.

### Exposure to Violence

Students were asked to respond “yes” or “no” to two questions: “Have you been exposed to any threats or threats of violence during the past twelve months that were so dangerous or serious that they scared you?” and “Have you been a victim of physical violence at any time during the last twelve months?” These two questions have been widely used in public health surveys e.g. [49] and are regarded as targeting exposure to psychological violence and physical violence, respectively.

### Sexual Coercion

The questions employed to assess the experience of sexual coercion ever in lifetime were drawn from an instrument that had been used in a large population study on sexual behavior in Sweden. In a previous study we found that 31% of the current study population had experienced sexual coercion [8]. We did not base the definition of sexual coercion on any particular theory of power relations but sought to make it as straightforward as possible. The definition of “being forced to do something”, i.e., to participate in a sexual situation, actively or passively was left to the respondent in the belief that the question would be reasonably well understood on a general operational level. Thus, the experience of sexual coercion was based on a response of “yes” to any of the following questions: “You have been forced to show your sexual organ”, “Someone has forced you to let them touch your sexual organ”, “Someone forced you to let them suck or lick your sexual organ”, “Someone has forced you to let them show you their sexual organ”, “You have been forced to watch someone masturbate”, “You have been forced to masturbate someone”, “You have been forced to take part in oral sex or to lick someone’s sexual organ”, “You have been forced to take part in sexual intercourse with the penis in the vagina, or someone has inserted an object into your vagina”, or “You have been forced to pose for a sex photo or sex film”. An individual was classified as not exposed to sexual coercion in the absence of an affirmative answer to any of the above questions, and with an affirmative answer to the question “You have not been forced into any of these”.

### Mental Health

Mental health status was assessed using the Hopkins Symptom Checklist (HSCL-25) [50], which assesses symptoms of anxiety and depression during the preceding week. This instrument contains 15 items reflecting depression and 10 items reflecting anxiety, rated on a scale from 1 (“not at all”) to 4 (“extremely”). In addition, 10 items from the Symptom Checklist-90 (SCL-90) sub-scale [51] reflecting psychoticism were included. These items primarily assess thought disturbances and perceptions that something is wrong with one’s body or mind. The SCL-90 is a self-report instrument for the assessment of psychiatric symptoms on a scale of 0 to 4. In order to simplify the responses, the 10 psychoticism items and the anxiety and depression items were both rated on a scale of 1 to 4. For each item students were asked, “How much this problem has bothered or distressed you during the last week, counting today?” Both the HSCL-25 and the SCL-90 have shown reliability and validity in a wide variety of cultural contexts [50], [52], including a population-based study in the same region of Uganda in which our investigation was conducted [53].

Because the HSCL-25 and the SCL-90 items could be summarized as individual mean scores, a standardized mean score for anxiety, depression, psychoticism, and total mental health symptom scores was obtained for each respondent. This was based on the student’s total score for that measure divided by the number of items answered. For the purpose of analysis, a dichotomized measure representing “high” (i.e. poor mental health status) versus “low” (i.e. satisfactory mental health status) summary scores was created separately by gender, based on a median-split of the distributions of the summary scores for each measure. A similar procedure has been used previously in a Ugandan setting [52].

### Data Analysis

Statistical analysis was performed using SPSS Version 16. Chi-square tests were used for the assessment of group differences in categorical variables. Independent t-tests were used for the assessment of gender differences in age and in mean scores of anxiety, depression, and psychoticism. Logistic regression was used to examine the association between sexual coercion and exposure to violence (both threatened and actual) and between sexual coercion, exposure to violence and poor mental health, with total symptom score as the dependent variable. Crude odds ratios (OR) and 95% confidence intervals (CI) for the experience of sexual coercion were adjusted for age. For poor mental health status, the crude OR and 95% CI were adjusted stepwise for socio-demographic and lifestyle characteristics, i.e., age, gender, alcohol consumption, smoking cannabis, area of origin and educational level of the head of household during upbringing. Statistical significance was accepted at *p*<0.05.

## Results

A total of 980 students filled out the questionnaire, representing 80% of the registered students at the time of the study (633 [64.6%] males and 347 [35.4%] females). The mean age of the study population was 23.3 years (SD = 3.5).

Table 1 shows the distribution of social and demographic background characteristics for the total sample and separately by gender. Females were younger than males. A significantly larger percentage of females (69.0%) than males (31.0%) grew up in an urban or semi-urban setting (*p*<0.001). More females than males grew up in households whose head had a medium or high education level (*p*<0.001). Women reported less use of cannabis during the past year and over their lifetime than men (*p*<0.01), and more women than men reported that they never drank alcohol (*p*<0.001).

**Table 1:** Prevalence of socio-demographic factors, smoking cannabis, and consumption of alcohol among 980 university students in south-western Uganda.

Variables	Totaln (%)	Malen (%)	Femalen (%)	*p* value
*Sex*Male*Age*	633 (64.6)347 (35.4)			
Younger (≤23years)	628 (65.6)	378 (60.6)	250 (75.1)	<0.01
Older (>23years)	329 (34.4)	246 (39.4)	83 (24.9)	
Missing	(23)	(9)	(14)	
*Area of origin*				
Urban/semi-urban	546 (56.3)	310 (49.4)	236 (69.0)	<0.001
Rural	424 (43.7)	318 (50.6)	106 (31.0)	
Missing	(10)	(5)	(5)	
*Educational level of head of household*				
High	473 (51.3)	279 (46.5)	194 (60.1)	<0.001
Medium	340 (36.8)	233 (38.8)	107 (33.1)	
Low	110 (11.9)	88 (14.7)	22 (6.8)	
Missing	(57)	(33)	(24)	
*Smoked cannabis*				
No	822 (93.4)	530 (91.9)	292 (96.4)	<0.01
Yes, in past month	24 (2.7)	16 (2.8)	8 (2.6)	
Yes, in the past yearor more	34 (3.9)	33 (5.7)	3 (1.0)	
Missing	(100)	(56)	(44)	
*Consumption of alcohol*				
Never	496 (53.7)	300 (50.4)	198 (11.1)	<0.001
Seldom	324 (35.1)	218 (36.6)	89 (28.4)	
Frequently	103 (10.2)	77 (12.9)	26 (8.5)	
Missing	(57)	(23)	(34)	

### Prevalence of Sexual Coercion, Threats/threats of Violence, and Actual Violence

Of the total sample, 31.1% had experienced some form of sexual coercion in their lifetime, with no significant gender differences. In the total sample, 27.8% of the respondents reported exposure to perceived threats/threats of violence inciting fear and 9.6% to actual physical violence during the past year (Table 2). There were no significant gender differences in exposure to perceived threats/threats of violence or actual physical violence.

**Table 2:** Exposure to perceived threat of violence and actual physical violence among 980 university students in south-western Uganda.

Category of violence	TotalN (%)	MaleN %)	FemaleN %)	*p-*value
*Exposed to threats of violence during the previous 12 months*				
No	689 (72.2)	440 (71.3)	249 (73.9)	
Yes	265 (27.8)	177 (28.7)	88 (26.1)	0.40
Missing	(26)	(16)	(10)	
*Exposed to physical violence during the previous 12 months*				
No	861 (90.4)	550 (89.3)	311 (92.6)	
Yes	91 (9.6)	66 (10.7)	25 (7.4)	0.10
Missing	28	(17)	(11)	

### Relationship between Exposure to Violence and Experience of Sexual Coercion

Table 3 provides an analysis of the associations between the perceived exposure in the previous 12 months to threats/threats of violence or physical violence and perceived exposure to sexual coercion ever in lifetime. The results show that the perceived exposure to threats/threats of violence or physical violence was significantly associated with experience of sexual coercion (OR _adjusted_ 2.2, 95% CI 1.6–3.0; OR_ adjusted_ 1.7, 95% CI 1.03–2.8, respectively). The association between perceived threats/threats of violence and students’ experience of sexual coercion was stronger in females than males (OR _adjusted_ 2.4, 95% CI 1.4–4.2); OR_ adjusted_ 2.1, 95% CI 1.4–3.2, respectively). The strength of the association between physical violence and the experience of sexual coercion remained unchanged but was non-significant after stratifying by sex.

**Table 3:** Relationship between exposure to violence and experience of sexual coercion among 980 university students in south-western Uganda in 2005 (logistic regression).

Experience of sexual coercion, crude OR (95% CI)			
	All	Females	Males
*Threat of violence*			
No	1.0 (ref)	1.0 (ref)	1.0 (ref)
Yes	2.2 (1.6–3.1)	2.6 (1.1–4.5)	2.1 (1.4–3.1)
*Physical violence*			
No	1.0 (ref)	1.0 (ref)	1.0 (ref)
Yes	1.7 (1.1–2.8)	1.9 (0.8–4.7)	1.7 (0.96–3.1)
**Experience of sexual coercion, adjusted OR (95% CI)**			
	**All**	**Females**	**Males**
*Threat of violence*			
No	1.0 (ref)	1.0 (ref)	1.0 (ref)
Yes	2.2 (1.6–3.0)	2.4 (1.4–4.2)	2.1 (1.4–3.2)
*Physical violence*			
No	1.0 (ref)	1.0 (ref)	1.0 (ref)
Yes	1.7 (1.03–2.8)	1.7 (0.7–4.4)	1.7 (0.9–3.0)

### Relationship between Exposure to Violence and High Symptom Scores (Poor Mental Health)

In a previous study of the same study population, we found that females and males had similar mean scores on all symptom sub- scales for mental health [46].

Table 4 shows the results for the relationship between symptom scores and exposure to sexual coercion and violence, respectively. Exposure to sexual coercion ever in lifetime was significantly associated with high anxiety scores in both females and males (p<0.001, respectively), high depression scores in both females and males (p<0.001, respectively), high psychoticism scores in both females and males (p<0.001, respectively), and high total symptom scores (p<0.001, respectively). Perceived exposure to threats/threats of violence in the previous 12 months was significantly associated with high anxiety scores in both females and males (*p* = 0.02, *p*<0.001, respectively), whereas exposure to physical violence was significantly associated with high scores of anxiety in males (*p* = 0.02) but not in females. In both males and females, perceived exposure to threats/threats of violence was significantly associated with high scores for depression, (*p* = 0.004 and *p*<0.001, respectively). The relationship between exposure to physical violence and high depression scores was significant only in males (*p*<0.001). Perceived exposure to threats/threats of violence was significantly related to high scores of psychoticism in both females and males (*p* = 0.02 and *p*<0.001, respectively). Exposure to physical violence was significantly associated with high scores in psychoticism in males only (*p* = 0.002). Both females and males showed a significant association between threats/threats of violence and high total symptom scores (*p* = 0.02 and *p*<0.001, respectively). However, only males showed a strong association between exposure to physical violence and total symptom scores (*p*<0.001).

**Table 4:** Relationship between exposure to violence and high vs. low scores on anxiety, depression, psychoticism, and total mental health symptom scores among 980 university students in south-western Uganda.

	Anxiety scores		Depression scores		Psychoticism scores		Total mental health scores	
**Females**	High	Low	High	Low	High	Low	High	Low
*Experience of sexual coercion*								
No	75 (54.0)	107 (80.5)	71 (52.6)	114 (79.7)	67 (50.4)	116 (82.3)	77 (53.1)	109 (81.3)
Yes	64 (46.0)	26 (19.5)	64 (47.4)	29 (20.3)	66 (49.6)	25 (17.7)	68 (46.9)	25 (18.7)
*p*-value*	<0.001		<0.001		<0.001		<0.001	
*Perceived threat of violence*								
No	111 (69.8)	129 (81.1)	104 (66.7)	137 (80.6)	109 (69.4)	133 (80.6)	116 (68.6)	127(79.9)
Yes	48 (30.2)	30 (18.9)	52 (33.3)	33 (19.4)	48 (30.6)	32 (19.4)	53 (31.4)	32 (20.1)
*p*-value	<0.05		<0.001		<0.05			
*Exposure to physical violence*								
No	147 (91.9)	149 (94.3)	142 (91.0)	159 (94.1)	145 (92.4)	153 (92.3)	155 (91.7)	148 (93.7)
Yes	13 (8.1)	9 (5.7)	14 (9.0)	10 (5.9)	12 (7.6)	11 (6.7)	14 (8.3)	10 (6.3)
*p-*value*							<0.05	
**Males**								
*Experience of sexual coercion*								
No	137 (58.3)	197 (78.8)	156 (59.3)	191 (81.3)	149 (60.1)	190 (78.8)	155 (59.8)	193 (80.4)
Yes	98 (41.7)	53 (21.2)	107 (40.7)	44 (18.7)	99 (39.9)	51 (21.2)	104 (40.2)	47 (19.6)
*p*-value*	<0.001		<0.001		<0.001		<0.001	
*Perceived threat of violence*								
No	163 (60.4)	250 (80.4)	187 (63.2)	236 (78.9)	166 (59.3)	247 (81.3)	176 (60.1)	247 (81.2)
Yes	107 (39.6)	61 (19.6)	109 (36.8)	63 (21.1)	114 (40.7)	57 (18.7)	117 (39.9)	57 (18.8)
*p-*value*	<0.01		<0.01		<0.001	<0.01		
*Exposure to physical violence*								
No	230 (85.8)	287 (92.0)	249 (84.4)	281 (94.0)	237 (84.9)	283 (93.1)	247 (84.6)	285 (93.7)
Yes	38 (14.2)	25 (8.0)	46 (15.6)	18 (6.0)	42 (15.1)	21 (6.9)	45 (18.2)	19 (6.3)
*p-*value*	<0.05		<0.01				<0.01	

* *p*-value indicate group differences between high and low scores.

### Relationship between Exposure to Sexual Coercion, Violence and Poor Mental Health, Adjusted for Potential Covariates

Table 5 shows the relationship between exposure to sexual coercion, perceived threats/threats of violence, and actual violence, and poor mental health status, as represented by the measure “total mental health symptom score”, i.e., all 35 items representing anxiety, depression, and psychoticism. Total mental health score was dichotomized as “high” versus “low” scores. Only crude and fully adjusted models are shown. The association between exposure to sexual coercion and high total mental health scores, albeit slightly reduced after adjustment for age, gender, childhood residence, educational level of head of household, and alcohol and cannabis consumption, remained statistically significant in the final model (OR 2.6, 95% CI 1.84–3.80). The associations between exposure to threats/threats of violence and exposure to physical violence and high total mental health scores (poor mental health) were only marginally altered after similar adjustment and remained statistically significant in the final models (OR 2.6, 95% CI 1.89–3.70, OR 2.2, 95% CI 1.27–3.91, respectively) (Table 5). In all final models (see Table 5), although frequent alcohol consumption was independently associated with high scores of “mental health symptoms”, the association did not significantly modify the relationship between exposure to violence and mental health status.

**Table 5:** Association between; experience of sexual coercion, exposure to perceived threat of violence, physical violence, and high total mental health symptom scores among 980 university students in south-western Uganda.

Exposure Variables	Crude OR (95%CI)	Model 6 Adjusted OR* (95%CI)
**Sexual coercion**		
*Experience of sexual coercion*		
No	1 (ref)	1 (ref)
Yes	3.1 (2.2–4.3)	2.6 (1.8–3.8)
*Educational level of head of household*		
High	1 (ref)	1 (ref)
Medium	1.1 (0.8–1.6)	1.2 (0.8–1.9)
Low	1.3 (0.9–1.7)	1.3 (0.9–2.0)
*Alcohol consumption*		
Never	1 (ref)	1 (ref)
Seldom	1.2 (0.9–1.6)	1.3 (0.9–1.8)
Frequently	2.1 (1.3–3.2)	1.8 (1.0–3.2)
*Smoked cannabis*		
No	1 (ref)	1 (ref)
Yes, in past month	3.9 (1.4–10.5)	3.3 (0.9–12.2)
Yes, in the past year or more	1.7 (0.8–3.4)	1.0 (0.4–2.3)
**Threat of Violence**		
*Perceived threat of violence*		
No	1 (ref)	1 (ref)
Yes	2.6 (1.9–3.6)	2.6 (1.9–3.7)
*Educational level of head of household*		
High	1 (ref)	1 (Ref)
Medium	1.1 (0.8–1.6)	1.3 (0.8–2.2)
Low	1.3 (0.9–1.7)	1.4 (0.97–1.9)
*Alcohol consumption*		
Never	1 (ref)	1 (ref)
Seldom	1.2 (0.9–1.6)	1.3 (0.9–1.8)
Frequently	2.1 (1.3–3.2)	2.2 (1.3–3.7)
*Smoked cannabis*		
No	1 (ref)	1 (ref)
Yes, in past month	3.9 (1.4–10.5)	3.1 (0.96–9.7)
Yes, in the past year or more	1.7 (0.8–3.4)	1.1 (0.5–2.4)
**Physical violence**		
*Exposure to physical violence*		
No	1 (ref)	1 (ref)
Yes	2.3 (1.5–4.5)	2.2 (1.3–3.9)
*Educational level of head of household*		
High	1 (ref)	1 (ref)
Medium	1.1 (0.8–1.6)	1.2 (0.8–2.1)
Low	1.3 (0.9–1.7)	1.3 (0.9–1.8)
*Alcohol consumption*		
Never	1 (ref)	1 (ref)
Seldom	1.2 (0.9–1.6)	1.2 (0.9–1.7)
Frequently	2.1 (1.3–3.2)	1.9 (1.1–3.2)
*Smoked cannabis*		
No	1 (ref)	1 (ref)
Yes, in past month	3.9 (1.4–10.5)	3.1 (0.98–9.6)
Yes, in the past year or more	1.7 (0.8–3.4)	1.1 (0.5–2.3)

* adjusted stepwise for Age, Sex, Area of origin, Educational level of head of household, Alcohol consumption and Cannabis smoking in Models 1–6 respectively.

## Discussion

A significant association was found between the experience of sexual coercion and exposure to violence in this sample of university students in Uganda. Also, 27.8% of the respondents reported experiences of being exposed to threats/threats of violence and 9.6% to actual physical violence, with similar exposure rates for both measures among females and males. However, some gender differences were observed in the relationship between exposure to violence and measures of anxiety, depression, and psychoticism. Poor mental health status in females was generally more related to threats/threats of violence while males were more affected by actual physical violence. In contrast, sexual coercion was significantly associated with all measures of poor mental health in both males and females.

It may be noted that those who had been exposed to threats/threats of violence or actual physical violence were about twice as likely to experience sexual coercion. This supports the conclusions of a study among women in eastern Uganda showing that those who had been exposed to physical violence were at about four times the risk of experiencing sexual coercion [27]. The latter report showing a stronger association between exposure to physical violence and experience of sexual coercion than currently found is likely attributable to differences in characteristics of the study populations. That study focused solely on intimate partner violence against women. The prevalence of physical violence and sexual coercion was higher in that population than in the current population studied (physical violence prevalence was 14% versus 9.6%, and sexual coercion 37.0% versus 31.1%, respectively). Evidence from the aforementioned study showed higher educational level for women to be a protective factor with regard to intimate partner violence, whereas rural residence emerged as a risk factor [27]. Our cohort comprised males and females with higher education levels and a larger proportion of urban or semi-urban residence than the population in the other study, offering a plausible explanation for the lower strength of association between violence and sexual coercion found in the current sample. The cross-sectional design of the current study does not allow for any conclusions regarding the causal direction of the association between sexual coercion and violence.

A study conducted in Iran found a similar association between exposure to physical violence and experience of sexual coercion [3]. Of the 66 males with a history of physical violence, 90.9% had experienced sexual coercion (*p*<0.001), and among females the prevalence was 95.4% (*p*<0.001). Our findings that threats/threats of violence are significantly associated with the risk of being exposed to sexual coercion among young people in Uganda suggest that accumulating exposure to violence may be of considerable concern. Long-term cumulative exposure to violence may have especially adverse consequences in terms of invisible suffering and poor physical and mental health. We therefore recommend that one also investigate young females and males who report threats of or actual physical violence for exposure to sexual coercion.

Contrary to a number of studies suggesting that females are more vulnerable to violence and the consequent mental health outcomes than men [22], [54], [55], our study agrees with a relatively smaller number of studies [56], [57] reporting that, when considering a broad violence exposure spectrum, males and females may not differ in exposure to violence although the expression of mental health symptoms may differ.

Men and women may experience different types of violence, with girls and women experiencing more sexual violence and men experiencing more physical aggression [54]. Thus, it is plausible that the overall prevalence of emotional and physical abuse is the same in men and women. However, as there were relatively fewer women than men in the present study, and since exposure to actual physical violence was less frequent for women (Table 2), it is possible that an association between exposure to physical violence and mental health symptoms in women was difficult to detect. We found that females had higher mean scores for depression than males, but the relationship between violence and depression was similar for both females and males, suggesting that there may be other determinants of depression in women that are beyond the scope of this study.

These findings also indicate that exposure to threats/threats of violence and to actual violence itself is significantly associated with mental health symptoms, irrespective of age, gender, and other socio-demographic and lifestyle characteristics (see Table 5). Our respondents were university students, the majority of whom come from a family background where the head of household had medium or high education qualifications. More than 50% grew up in urban or semi-urban residences (Table 1). This is not representative of the general Ugandan population where, according to the 2002 national census, only 14% live in urban settings and 8.9% of the population aged 20 to 24 years were enrolled in post-secondary (higher) education [58]. Therefore, our results may not be generalizable to the broader population in Uganda. Also, in the absence of previous studies on violence in this region, it is difficult to interpret the magnitude of the prevalence rates obtained in this sample. However, a study among university students of a similar age in Italy [59] reported comparable results, with 21.5% of the males and 31% of the females exposed to peer violence, and an even higher prevalence has been reported among Finnish students [60], where at least 42% had experienced physical violence and 39.1% the threat of violence. Although Uganda has had a history of internal conflict, the extent to which such history might have influenced the rates of violence reported by these students is entirely unknown.

### Methodological Considerations

To our knowledge this is the first study in Uganda (and possibly in sub-Saharan Africa) to explore the association between physical or threatened violence and sexual coercion among university students, as well as the prevalence of exposure to violence and its relation to mental health symptoms.

In addition, although our study design limits the ability to draw conclusions with regard to causality, the temporality between the exposure and outcome variables (i.e., exposure to violence in the previous 12 months and current mental health symptoms), suggests a possible cause and effect association.

Another strength of this study is that the anonymity of our respondents was fully protected, which may have encouraged truthful reporting. The study questionnaire was tested on a sample of students prior to the study and confirmed to be understandable and thus appropriate for self-administration.

However, the study had a number of limitations. Although the participation rate of 80% was satisfactory, some questions were left unanswered. Males and females had equivalent rates of missing data, although for the two items regarding threats and actual violence exposure missing data were relatively infrequent.

Secondly, the assessment of exposure to violence was limited to two questions. No information was obtained regarding the frequency or situational aspects of the violence reported; that is, nothing is known about where the violence took place, who the perpetrators were, or the severity/type of violence or threat to which the respondents were exposed. It is therefore unknown whether or not women in this study were more exposed to male-female violence or if males were exposed to male-male violence, as reported by Baker and colleagues [54]. Also, it is possible that some under-reporting of violence exposure may have occurred, and the inclusion of additional items pertaining to violence might have resulted in greater disclosure by the study participants. However, although the situational aspects are entirely unknown, the results indicate a strong association between exposure to violence and experience of sexual coercion as well as between exposure to violence and poor mental health. It may nevertheless be noted that mental health status in the current study was operationalized in terms of scores on selected psychiatric symptoms, and a more comprehensive investigation of mental health status might reveal other consequences. Also, high symptom scores do not necessarily denote the presence of a psychiatric disorder, nor do low levels indicate “psychiatric normality”. The results of the previous study on experience of sexual coercion and risky sexual behaviour also showed that satisfactory mental health seemed to buffer the effect of sexual coercion [8]. We controlled for confounding by the most important socio-demographic and lifestyle characteristics, but cannot exclude confounding by variables not measured in this study.

### Conclusions

The current study suggests that a) both males and females were generally equally exposed to violence, b) exposure to threat/threat of violence was associated with experience of sexual coercion, c) young females and males in Uganda showed similar mental health effects of exposure to violence, and d) the violence found in our study population may have long-term negative mental health implications. As it was not possible to identify the types of violence reported by these young people or to specify the perpetrators, further study of the specific situational aspects concerning violence exposure would be needed in order to design adequate prevention or intervention strategies. However, at the time of our survey, no policy or programme was in place to create awareness of risks of violence or sexual coercion among students at the university. Our findings may serve as a baseline for interventions and continuing research aimed at preventing violence or sexual coercion.
